# Integrative Analysis of Multimodal Omics Data

**DOI:** 10.1146/annurev-statistics-042424-113016

**Published:** 2025-10-01

**Authors:** Gen Li, Eric F. Lock

**Affiliations:** 1 Department of Biostatistics, University of Michigan, Ann Arbor, Michigan, USA;; 2 Division of Biostatistics and Health Data Science, University of Minnesota, Minneapolis, Minnesota, USA;

**Keywords:** multiomics, multiview, data fusion, integration, heterogeneity, statistical learning

## Abstract

With advancements in technology and the decreasing cost of data acquisition, high-throughput omics data have become increasingly prevalent in biomedical research. These data are often collected across multiple omics modalities at different molecular levels, offering a comprehensive perspective on underlying biological mechanisms. However, the multimodal nature of multiomics data presents unique and complex challenges for statistical analysis. In this article, we provide a comprehensive review of recent advancements in statistical methods for multiomics data integration. We discuss key topics in unsupervised learning (including dimension reduction, clustering, and network analysis), supervised learning (including regression, classification, and mediation analysis), and other areas. Finally, we highlight unresolved challenges and propose promising directions for future research to further advance the field.

## 1. INTRODUCTION

**Figure 1:**
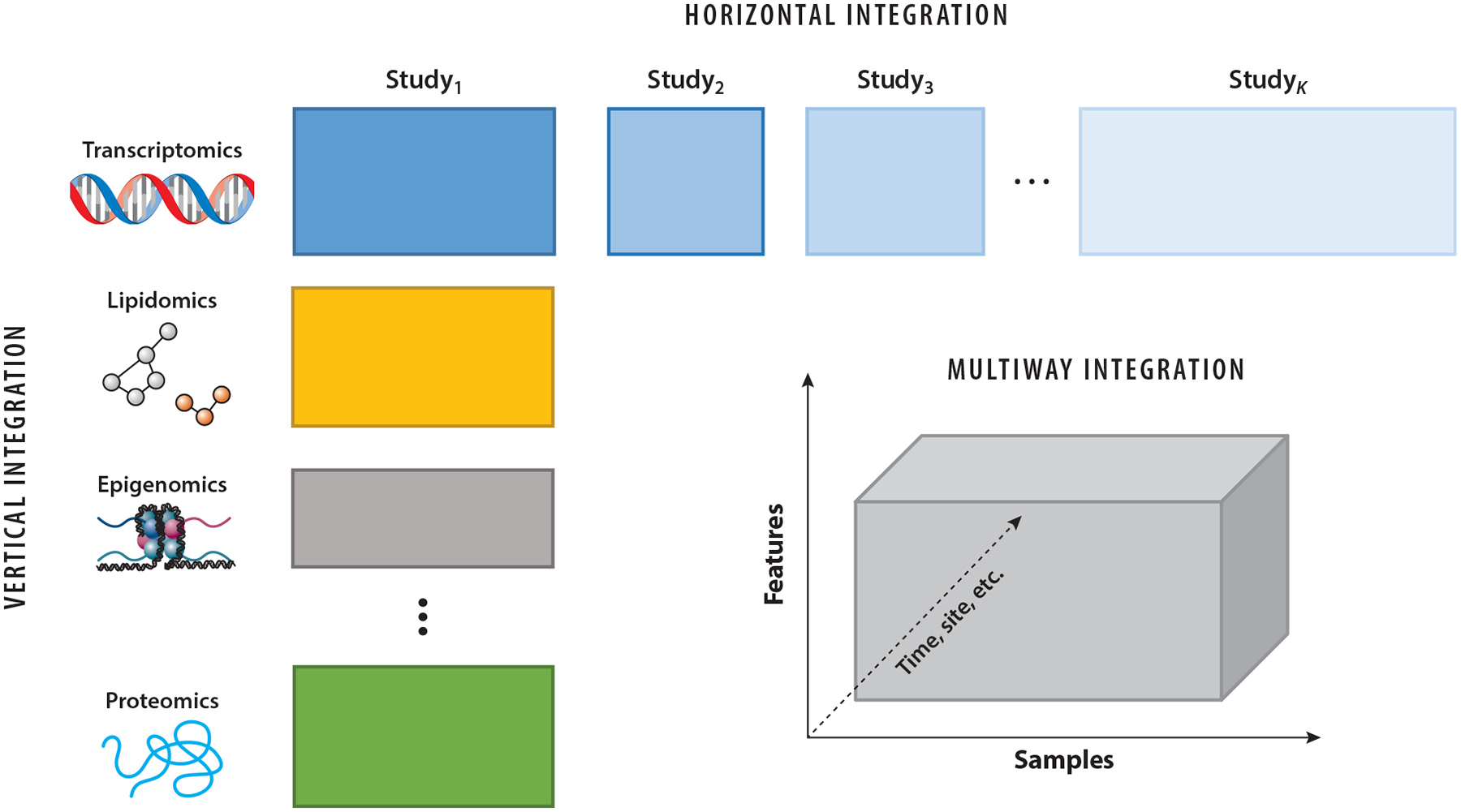
Illustration of multimodal omics data integration in biomedical research. Vertical integration refers to the integration of data from different omics platforms measured on the same set of samples. Horizontal integration involves combining data measured on the same set of features across multiple studies or cohorts. Multiway integration extends these frameworks to the same data collected repeatedly over other dimensions, such as time, different body sites, different tissues, or other conditions.

With rapid technological advancements and declining costs of data acquisition, multimodal (a.k.a. multiview) data have become increasingly prevalent in biomedical research. These data may be collected across various platforms, molecular levels, studies, or experimental conditions. As a result, integrating multiple data sources has become a central focus of modern biomedical data analysis. Depending on whether features, samples, or both are shared across datasets, integration strategies are commonly categorized into vertical integration, horizontal integration, and multiway integration (see Figure 1). This review primarily focuses on the vertical integration of different omics modalities measured on the same set of samples, with brief discussions of other integration types.

When collected across different omics platforms on matched samples, these data are referred to as multiomics data. Common omics modalities include genomics, transcriptomics, proteomics, metabolomics, lipidomics, and others. Historically, each omics layer was typically analyzed in isolation. However, this single-layer approach often provides limited and potentially biased insights, as most biological processes involve complex interactions across multiple molecular levels. In contrast, multiomics data offer a more comprehensive view of biological systems and enable deeper investigation into mechanistic pathways underlying health and disease (Karczewski & Snyder 2018).

In recent years, multiomics analyses have gained considerable traction. Empirical studies have demonstrated their value in elucidating biological mechanisms and disease etiology. A prominent example is the Cancer Genome Atlas (TCGA) project (Hutter & Zenklusen 2018), which characterized over 11,000 tumors across 33 cancer types using multiomics data, including genomics, transcriptomics, epigenomics, and proteomics. TCGA offered a more complete understanding of tumor biology, uncovering key regulatory mechanisms, pathway alterations, and clinically relevant subtypes that would have been missed using single-omics analyses alone (see, e.g., Cancer Genome Atlas Research Network 2012, 2014). Despite these successes, many existing analyses either naively concatenate omics datasets or integrate results from single-omics analyses post hoc, falling short of fully leveraging the richness of multiomics data.

The availability of multiomics data presents both unprecedented opportunities and significant methodological challenges. A growing body of literature has focused on developing statistical methods tailored to multiomics integration. Key challenges include heterogeneity in data types and distributions, complex interomic relationships, measurement errors, missing data patterns, and high computational demands. Different omics modalities typically vary in scale, dimension, and data structure. Interactions occur both within and across omics layers, often in complex, nonlinear ways. Additionally, due to technical limitations and cost constraints, omics profiles may be incomplete, resulting in block-wise missingness. The ultrahigh dimensionality of multiomics datasets also imposes substantial computational burdens.

“Data integration” is a broad term encompassing various analytical goals. Terms such as “multi-omics integration,” “multiview integration,” “multisource integration,” “multimodal integration,” and “data fusion” are often used interchangeably. In this review, we focus on widely studied statistical methodologies for multiomics integration within supervised and unsupervised learning frameworks. Specifically, we discuss joint data reduction, cross-omics network inference, integrative clustering, and between-omics association analysis in the context of unsupervised learning, as well as regression, classification, and mediation analysis in supervised learning. Our emphasis is on linear methods with clear probabilistic foundations, though we also briefly review nonlinear and deep learning (DL) approaches. Finally, we outline remaining methodological challenges and suggest several promising directions for future research.

Throughout the article, we use bold letters to denote matrices (e.g., X and 𝛀) and vectors (e.g., x and α). For multiomics data matrices, rows represent features and columns represent samples (i.e., X is a p×n matrix with p features and n samples). Different omics modalities are denoted using subscript indices (i.e., $X_{1},\ldots ,X_{K}$). Unless otherwise noted, we assume that samples are matched across modalities and that our focus remains on vertical integration of multiomics data.

## 2. UNSUPERVISED LEARNING

**Figure 2:**
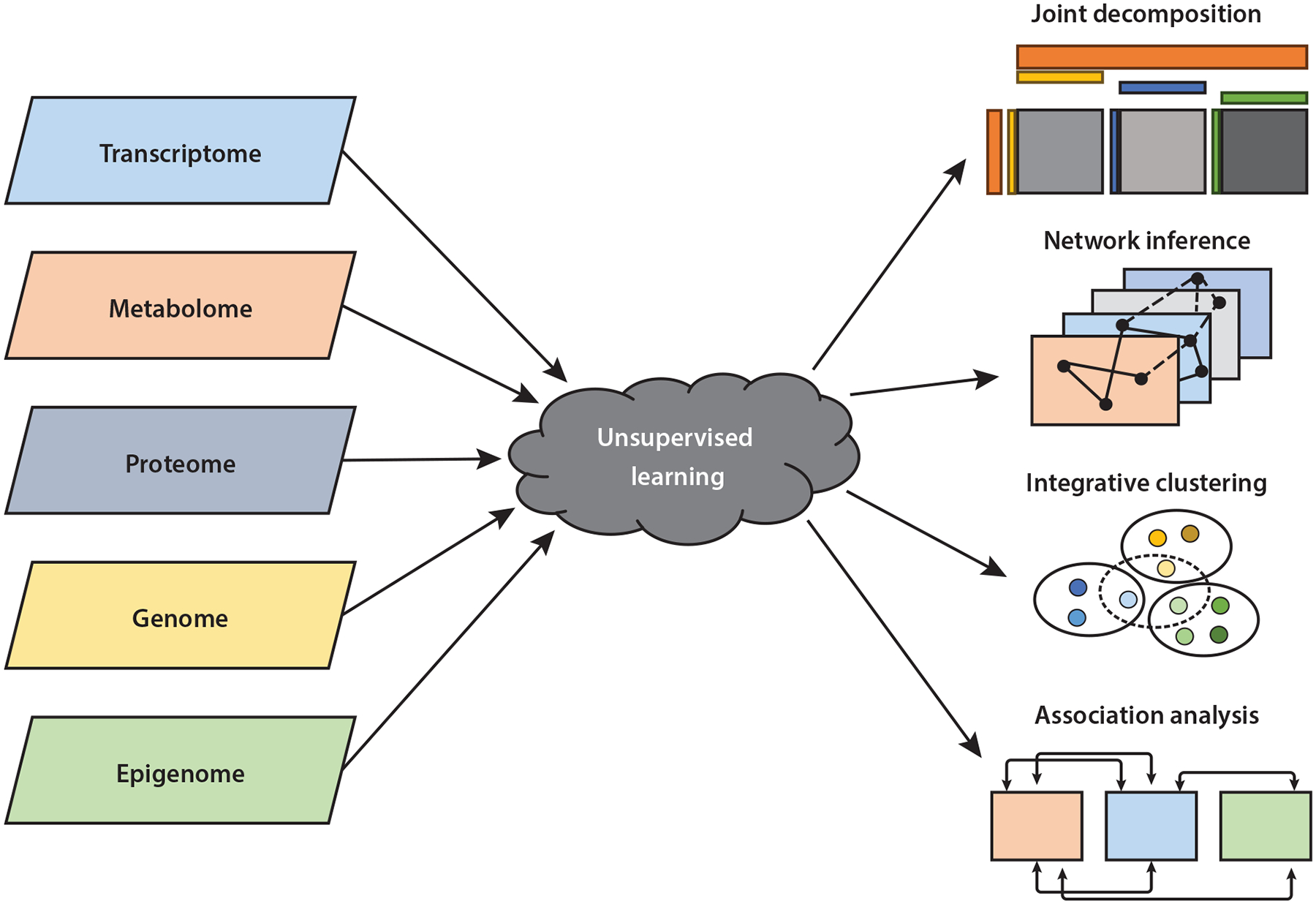
Illustration of key unsupervised learning topics in multiomics data integration, including joint decomposition of multiomics data, cross-omics network inference, integrative cluster analysis, and association analysis among omics modalities.

Unsupervised learning refers to a class of machine learning techniques that aim to uncover intrinsic patterns or structures in data without the use of labeled outcomes. It plays a critical role in tasks such as data denoising, dimension reduction, visualization, and subgroup identification, and it is particularly valuable for revealing latent structure in complex datasets and generating hypotheses for downstream investigation. In the context of multiomics data integration, key unsupervised learning tasks include joint dimension reduction across multiple omics modalities, inference of within- and cross-omics network structures, integrative clustering of samples, and analysis of interomics associations (see Figure 2). In this section, we review statistical methods developed for each of these tasks, with an emphasis on approaches that account for the heterogeneity and high dimensionality inherent in multiomics data.

### 2.1. Joint Dimension Reduction

Dimension reduction is often a crucial step in the analysis of omics data. For a single dataset, well-known methods such as principal components analysis (PCA) (Hotelling 1933), multidimensional scaling (Hout et al. 2013), and t-distributed stochastic neighbor embedding (Van der Maaten & Hinton 2008) reduce the high-dimensional space of the original features (genes, proteins, etc.) to a small number of latent patterns. For example, the first r principal components approximate a matrix $X\in R^{p\times n}(p\gg n)$ as X≈US, where $U\in R^{p\times r}$ are feature loadings with orthonormal columns and $S\in R^{r\times n}$ are sample scores. An r-dimensional space captures the majority of variation in the original p-dimensional space. The resulting scores can be used to visualize and interpret patterns of variability in the data, or as meta-variables in subsequent statistical models.

Dimension reduction for multiomics data has been a very active area of research in recent years. Methods such as consensus PCA (Westerhuis et al. 1998) identify latent components that are shared across multiple modalities $X_{1},\ldots ,X_{K}$. Often, we are interested not just in identifying associations between the modalities, but also in identifying the unique information in each. To this end, several methods have been developed that decompose lower-dimensional structure that is shared jointly among platforms or specific to each platform. The joint and individual variation explained (JIVE) method (Lock et al. 2013, O’Connell & Lock 2016) was developed as an extension of PCA for multiomics data:

$$X_{k}\approx U_{k}S+W_{k}S_{k}.$$

Joint structure is captured by the common score matrix $S\in R^{r\times n}$, while individual structure specific to each platform is captured by the individual score matrices $S_{k}\in R^{r_{k}\times n}$. A key observation motivating these methods is that accounting for systematic platform-specific variation improves detection of shared structure, and vice-versa. The decomposition is useful for interpreting shared or specific patterns of variation. The joint and individual scores may be included as meta-variables in subsequent predictive models, distinguishing concordant and complementary predictive information among the platforms (Kaplan & Lock 2017, Liu et al. 2024).

Multiomic decompositions of lower-dimensional structure have been enhanced and extended in several ways. Methods have been developed for diverse distributional forms in an exponential family framework (Li & Gaynanova 2018, Zhu et al. 2020), allowing the integration of count or binary data. Other methods allow for identification of partially shared structure among two or more modalities (Gaynanova & Li 2019, Yi et al. 2023). Yet other methods allow for covariates (e.g., clinical metrics) to inform the lower-dimensional components, leading to a more interpretable decomposition (Li & Jung 2017, Gao et al. 2021). Similar decompositions of shared and unshared structure have been proposed for nonnegative matrix factorization (Yang & Michailidis 2016), partial least squares (PLS) (el Bouhaddani et al. 2016), canonical correlation analysis (CCA) (Zhou et al. 2015), and nonparametric Bayesian factor models (Ray et al. 2014).

A key challenge for such multiomic decompositions of lower-dimensional structure is rank selection, i.e., determining the number of components that are shared or unshared across the modalities. For this, a wide variety of different strategies have been used, including permutation testing (Lock et al. 2013), the Bayesian information criterion (O’Connell & Lock 2016), cross-validation (Gaynanova & Li 2019, Yi et al. 2023), and angle-perturbation theory (Feng et al. 2018). Moreover, a wide range of estimation strategies and statistical frameworks have been considered, including least squares (Lock et al. 2013), penalized least squares (e.g., with $L_{1}$ or nuclear norm penalties) (Gaynanova & Li 2019, Yi et al. 2023), maximum likelihood (Li & Jung 2017), Bayesian objective functions (Argelaguet et al. 2018, Chekouo & Safo 2023), and fully Bayesian methods (Samorodnitsky et al. 2024).

Sandri et al. (2018) present an example of successful dimension reduction for multiomics analysis, using JIVE to integrate proteomic, cytoplasmic transcriptomic, and polyribosomal translatomic data from lung tissues of chronic obstructive pulmonary disease patients with and without lung cancer. JIVE effectively reduced the complexity of the combined datasets, identifying eight interpretable latent patterns—two joint, three individual to the transcriptome, and three individual to the translatome. Follow-up multinomial logistic regression for the eight components robustly distinguished tumor, adjacent nonmalignant, and control tissues. Notably, the integrated analysis revealed that transcriptomic signals predominated in distinguishing tumor tissue, while translatomic signals were key in differentiating tumor-adjacent stroma from controls. This dimensionality reduction not only simplified the multilayered omics data but also enabled the discovery of relevant pathways via enrichment analysis of the component loadings, such as extracellular matrix and PI3K-Akt signaling, implicated in tumor-supportive stromal environments.

### 2.2. Network Inference

Network inference enables researchers to investigate dependence relationships among features. The typical output is a network or graph, where nodes represent features and edges represent interactions between them. These edges are often weighted, and in some cases directed, with the weight reflecting the strength of interaction and the direction indicating potential regulatory relationships. In single-modality data analysis, commonly used approaches include correlation-based methods such as thresholded correlation estimation and partial-correlation-based methods such as graphical models. While correlation-based methods are computationally straightforward, they tend to produce dense and difficult-to-interpret networks. In contrast, partial-correlation-based methods focus on conditional independence and are thus better suited to capturing the underlying dependence structure, making them more widely used in practice.

In the context of multiomics data, network inference becomes more complex, as it involves integrating information across different omics modalities. Research questions in this setting generally fall into three categories. The first category focuses on characterizing the dependence structure within a primary modality while adjusting for auxiliary omics data. For example, one might be interested in gene expression networks, using other modalities such as methylation or proteomics as covariates. This problem is typically framed as a multivariate regression model, where the objective is to estimate the precision matrix of the response variables after accounting for covariate effects. Maximum likelihood estimation is commonly employed, with sparsity-inducing penalties to prevent overfitting and encourage interpretable networks (Rothman et al. 2010, Lee & Liu 2012, Cai et al. 2013). Building on this framework, Lin et al. (2016) and Majumdar & Michailidis (2022) proposed multilayered graphical models that extend the regression formulation to account for unidirectional regulatory relationships across omics layers. More recently, Zhang & Li (2023) developed graphical regression models that allow the network structure to vary with external covariates.

The second category aims to identify shared and modality-specific network structures across multiple omics datasets measured on the same set of features. For instance, one might analyze gene–gene networks inferred from both gene expression and methylation data. Although the feature set remains the same, each modality may capture different aspects of the dependence structure. The goal is to extract common network patterns while isolating modality-specific effects. Guo et al. (2011) introduced a joint Gaussian likelihood framework in which the network structures are encoded by reparameterized precision matrices, decomposed into shared and specific components. This idea has been further developed through alternative parameterizations and regularization techniques. For example, Danaher et al. (2014) proposed models for smoothly varying network structures, Zhu et al. (2014) introduced entry-wise clustering across networks, and Bayesian approaches have been explored to incorporate prior knowledge and encourage structured shrinkage (Peterson et al. 2015, Ma & Michailidis 2016, Saegusa & Shojaie 2016). More recently, Li & Wang (2024) proposed a method for joint estimation of multiple network structures while simultaneously clustering the modalities based on structural similarities. The method was applied to the National Institutes of Health Common Fund’s Genotype-Tissue Expression (GTEx) project gene expression data in brain tissues to identify gene coexpression networks and brain tissue clusters.

The third category focuses on interactions between features across different modalities, rather than within individual omics layers. For example, researchers may aim to identify microbiome–metabolite pairs in the gut that regulate host metabolism, or investigate transcript–microbiome–metabolite interactions relevant to asthma treatment. Accurate modeling of such cross-modality interactions requires adjusting for within-modality correlations and potential confounders. A common approach is to concatenate features from different datasets into a single matrix and apply standard graphical modeling techniques. While this method is conceptually simple, it can become computationally intensive and statistically inefficient due to the ultrahigh dimensionality of the combined data. To address this, Sohn & Kim (2012) and Yuan & Zhang (2014) developed conditional graphical models that provide a more structured approach to cross-modality network estimation in two datasets. Rather than modeling the full joint distribution of the two datasets, these methods estimate the conditional precision matrix of one modality given another, enabling scalable inference without requiring assumptions or estimation for the full network structure. This approach captures conditional dependence across modalities while accounting for within-modality correlations, and it has been studied in a Bayesian framework as well (Chiquet et al. 2017).

### 2.3. Integrative Clustering

Multiomics data exhibit substantial heterogeneity across biological samples and individuals. Clustering methods aid in understanding this heterogeneity by grouping samples with similar molecular profiles. This, in turn, enables researchers to identify novel subpopulations or disease subtypes.

To identify shared subtypes across multiple modalities, a popular approach is the iCluster framework (Shen et al. 2009). iCluster identifies latent factors shared across modalities, analogous to consensus PCA in Section 2.1; then, clusters are identified from the estimated factors using traditional approaches such as k-means clustering. The original iCluster method has been extended to accommodate discrete and continuous modalities (Mo et al. 2013) and fully Bayesian inference (Mo et al. 2018). Hellton & Thoresen (2016) apply k-means clustering on latent factors identified by JIVE (see Section 2.1) to identify joint clusters or individual clusters for each modality, given by clustering of the joint and individual structure, respectively.

Another common strategy is to allow a distinct clustering of the sample set for each modality, while modeling their dependence or consensus. Kirk et al. (2012) describe a Bayesian approach to cluster each modality with a general dependence model between cluster memberships across modalities. Lock & Dunson (2013) describe Bayesian consensus clustering, in which modality-specific clusterings are shrunk toward an overall clustering of the samples that are inferred together in a Bayesian framework. The Clusternomics method (Gabasova et al. 2017) uses a similar but more flexible framework to allow for multiple shared clusterings using a hierarchal Dirichlet process prior.

Yet another common approach is to combine similarity or distance matrices across modalities to inform clustering. Similarity network fusion (Wang et al. 2014) is a popular approach to combine similarity networks for the samples in each modality; the fused similarity networks may be used to find shared clusters. Multikernel learning (Speicher & Pfeifer 2015) combines distance kernels across modalities (potentially with multiple kernels per modality) to identify shared clusters. An advantage of such approaches is that they naturally accommodate heterogeneity across modalities via different distance or similarity metrics.

Numerous studies have applied integrative clustering to define subtypes in different disease areas. For example, Shen et al. (2012) applied iCluster across copy number, DNA methylation, and gene expression data from glioblastoma multiforme samples. They identified three well-defined subtypes of glioblastoma multiforme: one lacking chromosome 7 gain/chromosome 10 loss, enriched for G-CIMP (glioma CpG island methylator phenotype) and proneural expression; a second defined by EGFR (epidermal growth factor receptor) amplification, promoter methylation of homeobox and G-protein signaling genes, and a classical expression profile; and a third characterized by NF1 (neurofibromatosis type 1) and PTEN (phosphatase and tensin homolog) alterations with a mesenchymal-like expression pattern.

### 2.4. Association Analysis

Another common task in unsupervised learning for multimodal omics data is the association analysis between multiple datasets, which can be performed in either a symmetric or an asymmetric manner (Huang et al. 2017, Csala & Zwinderman 2019). Symmetric approaches focus on uncovering shared correlation or covariance structures without imposing directional or causal assumptions. In contrast, asymmetric methods model directed relationships between omics modalities, often informed by biological hierarchies such as the central dogma of molecular biology (DNA is transcribed into mRNA, which is translated into proteins). Symmetric methods include CCA, PLS, coinertia analysis (CIA), and their various extensions. Asymmetric methods encompass redundancy analysis (RDA), reduced rank regression (RRR), envelope models, and other multivariate regression techniques. Association analysis of this kind has the potential to uncover complex biological pathways and regulatory mechanisms across multiple omics layers.

Formally, let $X_{1}\in R^{p_{1}\times n}$ and $X_{2}\in R^{p_{2}\times n}$ denote two centered data matrices measured on the same set of n samples. CCA seeks loading vectors α and β that maximize the correlation between the linear projections of the two datasets:

$${\text{argmax}}_{\alpha ,\beta }\;\text{Corr}\left(X_{1}^{⊤}\alpha ,X_{2}^{⊤}\beta \right),$$

subject to appropriate norm constraints. Subsequent pairs of canonical directions are obtained by constraining the search to the orthogonal complement of the previous ones. While CCA is effective in low-dimensional settings, its reliance on inverting sample covariance matrices becomes problematic in high dimensions. To address this, numerous regularized extensions have been proposed that simultaneously select relevant variables and maximize canonical correlations (Tenenhaus & Tenenhaus 2011, Chen et al. 2013, Lin et al. 2013, Mai & Zhang 2019, Wróbel et al. 2024).

PLS offers an alternative by maximizing the covariance, rather than the correlation, between the projections of the two datasets. Similar to CCA, PLS has been extended to high-dimensional settings through sparse and penalized formulations (Lê Cao et al. 2008, Chun & Keleş 2009, el Bouhaddani et al. 2016). CIA is closely related to CCA and PLS but further introduces adaptive weights for both features and samples, thereby enhancing flexibility in capturing costructure between datasets (Dolédec & Chessel 1994). For instance, Min et al. (2019) utilized a penalized CIA method to integrate the gene expression and protein abundance data in the NCI-60 cancer cell line and identified many biologically meaningful pathways enriched in various cancers.

When a directional relationship is known, such as $X_{1}\to X_{2}$, asymmetric methods are often more appropriate. These approaches aim to identify linear combinations of explanatory variables in $X_{1}$ that explain maximal variance in response variables $X_{2}$. RDA is a family of algorithmic procedures that generally involve performing dimension reduction following multivariate linear regression (Borcard et al. 2011, Csala et al. 2017). Model-based alternatives include RRR (Izenman 1975, Reinsel & Velu 1998) and envelope models (Cook et al. 2010, Cook & Zhang 2015), both of which decompose the variation in $X_{2}$ into a component explained by $X_{1}$ and an immaterial component. An in-depth review of recent developments in envelope methods is provided by Lee & Su (2020).

While most conventional methods are designed for two datasets, extensions to multiple omics layers have been proposed. For example, Witten & Tibshirani (2009) generalized CCA to accommodate more than two datasets, and Csala et al. (2019) extended RDA using PLS path modeling (Tenenhaus et al. 2005). Li et al. (2019) proposed an integrative RRR framework for multiset asymmetric association analysis.

## 3. SUPERVISED LEARNING

**Figure 3:**
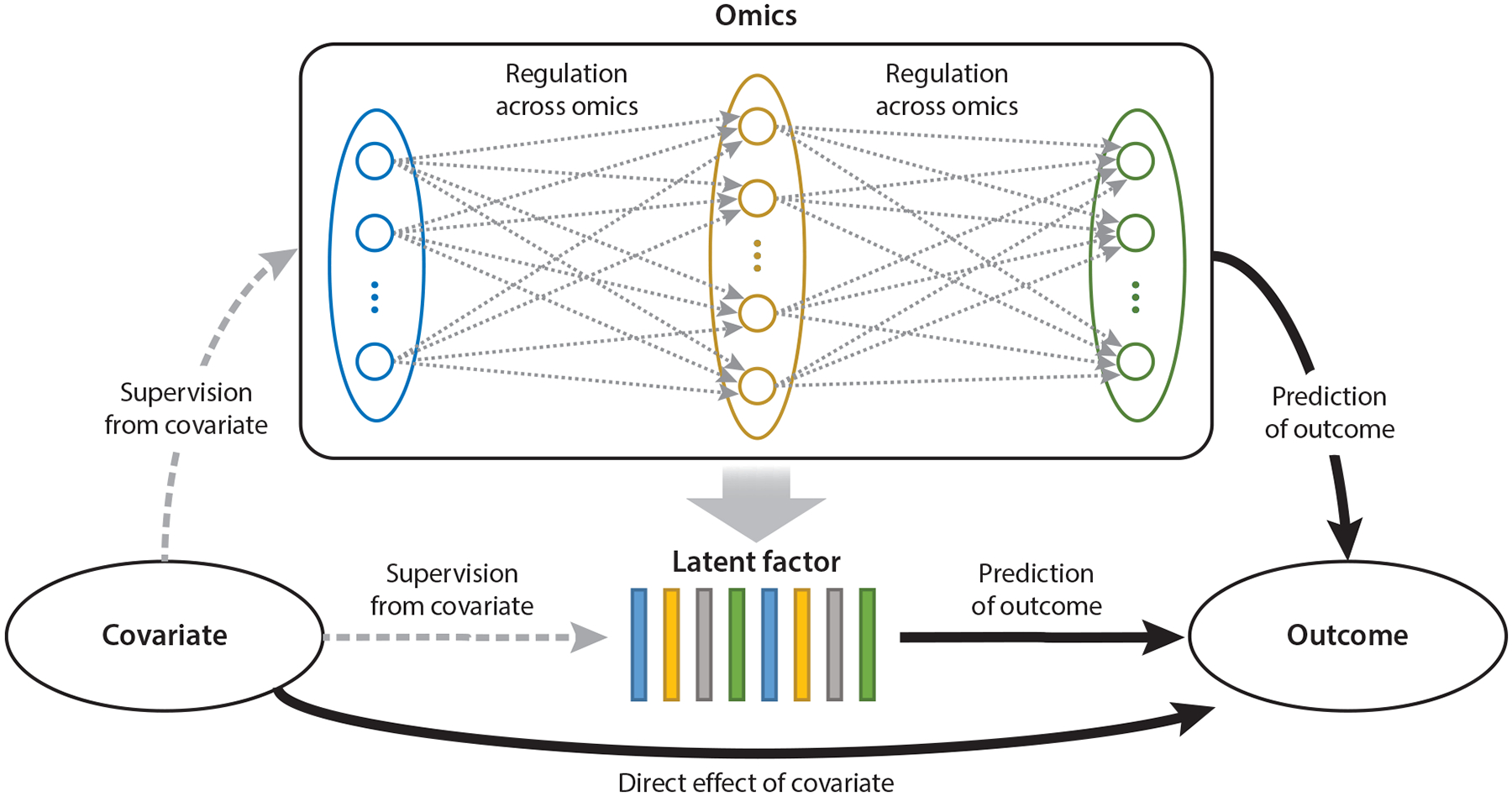
Schematic illustration of supervised learning in multiomics data integration. The diagram highlights several key topics: latent variable models where external covariates supervise the dimension reduction of multiomics data, predictive modeling frameworks where multiple omics modalities jointly serve as predictors of clinical outcomes, and mediation analysis that explores potential causal pathways from covariates to outcomes through intermediate omics layers.

Supervised learning refers to a class of machine learning methods that utilize labeled data to train models capable of predicting outcomes or classifying observations. In the context of multiomics data integration, supervised learning plays a pivotal role in identifying biologically meaningful patterns across heterogeneous omics layers that are associated with specific phenotypes or clinical outcomes. By modeling predictive relationships from high-dimensional, complex, and often noisy omics data, supervised learning approaches can facilitate biomarker discovery, disease subtype classification, and mechanistic understanding through mediation analysis. Successful application of supervised learning in multiomics settings requires careful handling of cross-modality regulatory relationships, complex correlation structures, and high dimensionality. In particular, effective feature selection and dimension reduction are essential to mitigate overfitting and enhance model interpretability (see Figure 3). In this section, we review key methodological developments in supervised learning for multiomics data, focusing on regression, classification, and mediation analysis.

### 3.1. Integrative Regression Analysis

When the primary objective is to predict a quantitative outcome $y\in R^{n}$ using multiomics data, integrative regression models are commonly employed. Regression analysis in this context not only supports predictive modeling and decision-making but also aids in identifying important omics features associated with clinical outcomes. Assuming that multiple omics modalities are measured on the same set of samples, a naive strategy is to concatenate all datasets into a single matrix and apply standard regression techniques. However, this approach overlooks the intrinsic heterogeneity across modalities, such as differences in scale, dimensionality, and data type, and fails to account for known biological or structural relationships between omics layers. Furthermore, issues such as high dimensionality, block-wise missingness, and multicollinearity pose serious challenges to the naive approach.

One way to address these challenges is to first reduce the dimensionality of the omics data prior to regression. Joint dimension reduction methods, as reviewed in Section 2.1, can be used to obtain low-rank representations of the multiomics data. For example, Kaplan & Lock (2017) proposed a two-step procedure in which the multiomics data are decomposed using JIVE, and the outcome is subsequently regressed on the resulting joint and individual scores. Building on this, Palzer et al. (2022) developed a one-step approach that simultaneously decomposes the omics data and fits the predictive model. By incorporating outcome information into the dimension reduction process, this method enhances predictive performance.

To facilitate variable selection in multiomics predictors, Li & Li (2021) proposed an integrative factor regression model that decomposes each data modality separately using factor analysis and then regresses the outcome on the resulting latent factors and idiosyncratic errors. Specifically, let y denote a univariate response and $X_{k}\in R^{p_{k}\times n}(k=1,\ldots ,K)$ denote the kth omics data matrix. Rather than directly estimating the regression coefficients in the linear model $y=\sum_{k=1}^{K}X_{k}^{⊤}\beta_{k}+\varepsilon$, the integrative model leverages a rank-$r_{k}$ factor model for each data modality $X_{k}=W_{k}S_{k}+E_{k}$ to rewrite the linear regression in the following equivalent form:

$$y=S^{⊤}\gamma^{⋆}+E^{⊤}\beta +\varepsilon ,$$

where $S={\left(S_{1}^{⊤},\ldots ,S_{K}^{⊤}\right)}^{⊤}\in R^{\left(r_{1}+\cdots +r_{K}\right)\times n}$ is the collection of individual factor scores, and $E={\left(E_{1}^{⊤},\ldots ,E_{K}^{⊤}\right)}^{⊤}\in R^{\left(p_{1}+\cdots +p_{K}\right)\times n}$ is the collection of idiosyncratic errors. The vector $\beta ={\left(\beta_{1}^{⊤},\ldots ,\beta_{K}^{⊤}\right)}^{⊤}$ represents the regression coefficients of primary interest, while $\gamma^{⋆}$ captures nuisance effects. This factor-based approach helps mitigate collinearity among omics features and improves both prediction accuracy and inference reliability (Li & Li 2021, Fan et al. 2020).

Another line of research further incorporates regulatory relationships between different omics layers directly into the regression framework. For instance, Wang et al. (2013) proposed the integrative Bayesian analysis of genomics data (iBAG), a gene-centric hierarchical model that includes two components: a mechanistic layer that models the regulation of transcript-level data by upstream omics (e.g., methylation) and a clinical layer that uses these components for outcome prediction. In the presence of gene expression data (G) and methylation data (M), the mechanistic model decomposes gene expression into a methylation-modulated component $G^{M}=𝚯M$ and a residual component $G^{\overset{‾}{M}}=G-G^{M}$ that is independent of methylation. The clinical model then regresses the outcome on both components. Model inference is carried out within a Bayesian framework using Markov chain Monte Carlo. A related approach was developed by Fang et al. (2018) to extend iBAG to settings with missing data across modalities. Such methods have been successfully used to integrate multiomics profiles, such as genotype, methylation, and gene expression data, to jointly predict cancer patient survival, which achieved superior prediction and interpretation (Bernal Rubio et al. 2018).

### 3.2. Classification

When the outcome of interest is binary $\left(y\in \{0,1{\}}^{n}\right)$ or belongs to multiple categories, the regression problem naturally transitions into a classification setting. In the context of omics data, classification is used to uncover biologically meaningful patterns and categorize samples based on molecular profiles—for example, distinguishing between diseased and healthy individuals or predicting treatment response. High-dimensional classification has been an active area of machine learning research, and various extensions have been proposed to accommodate multimodal omics integration.

Several methods incorporate supervision into multiomics association or dimension reduction frameworks to improve classification performance. Joint association and classification analysis (Zhang & Gaynanova 2022) and sparse integrative discriminant analysis (Safo et al. 2022) combine linear discriminant analysis with CCA to identify latent representations that are both correlated across modalities and discriminative for group membership. Related approaches have extended this framework to capture nonlinear relationships using DL (Safo & Lu 2024, Wang & Safo 2024). Bayesian integration and prediction (Chekouo & Safo 2023) employs a Bayesian factor model to jointly model multiomics data and predict a binary outcome using covariates. Similarly, Samorodnitsky et al. (2024) propose a Bayesian multiomics factor model in which a binary outcome is predicted via probit regression from both shared and modality-specific latent factors.

Other approaches perform classification directly, without relying on latent factor models, by extending classical high-dimensional classification techniques to the multiomics setting. Block-Forest (Hornung & Wright 2019) generalizes random forests (Breiman 2001) by allowing decision tree splits to incorporate features across multiple modalities. Kim et al. (2023) proposed a Bayesian linear classification framework that introduces modality-specific regularization through Gaussian priors, accommodating the diverse scales and structures of multiomics data; their approach identified a metabolomic and proteomic profile that provided perfect separation of anemic and nonanemic infants in early development in a rhesus monkey model. Ding et al. (2022) introduced a collaborative learning approach that bridges direct and latent-factor-based methods by including a penalty term that encourages consistent predictions across omics layers.

### 3.3. Mediation Analysis

Mediation analysis is a powerful tool for disentangling regulatory pathways between an exposure (e.g., treatment) and an outcome (e.g., survival). It decomposes the total effect of the exposure into an indirect effect, mediated through intermediate variables, and a direct effect not explained by the mediators. Classical approaches are typically limited to settings with a single or low-dimensional set of mediators. Commonly used frameworks include structural equation modeling (Baron & Kenny 1986, MacKinnon 2008) and counterfactual-based methods (Imai et al. 2010, Rubin 1974). Key objectives in mediation analysis include testing for the presence of mediation effects, quantifying their magnitude, and identifying specific mediators that significantly contribute to the pathway from exposure to outcome.

In multiomics research, mediation analysis faces two major challenges: the high dimensionality of omics features and the strong multicollinearity both within and across modalities (Hayes 2009, Blum et al. 2020). When mediators are high-dimensional, most existing approaches address the problem through dimensionality reduction or feature selection. Screening-based and penalization-based strategies have been widely adopted for mediator selection (Zhang et al. 2016, Zhao & Luo 2022), while dimension reduction techniques such as PCA have also been used to summarize omics signals (Huang & Pan 2016). Alternatively, gene-centric approaches focus on one gene at a time, identifying localized mediation signals (Fang et al. 2021). A comprehensive review of these strategies is provided by Zeng et al. (2021).

Methods specifically designed for multiomics mediation analysis are relatively limited. One line of research considers high-dimensional exposures and mediators simultaneously. For example, Zhang (2022), Long et al. (2020), and Zhao et al. (2022) proposed regularization-based approaches for path selection in high-dimensional linear mediation models. These methods have been applied to multiomics settings involving gene expression, protein abundance, and imaging-derived features, and identified meaningful regulatory pathways and mechanisms underlying complex diseases such as Alzheimer’s disease (Zhao 2024).

Another line of work addresses mediation analysis involving multiple sets of ordered mediators. These models are particularly relevant when omics layers follow a biologically meaningful order (e.g., epigenome → transcriptome → proteome). Daniel et al. (2015) proposed a counterfactual framework for decomposing effects through sequential mediators. Lai et al. (2020) extended this framework to binary outcomes, while Kidd et al. (2023) further developed methods to accommodate arbitrary missingness patterns across multiple mediator layers.

## 4. ADDITIONAL TOPICS

### 4.1. Horizontal and Bidimensional Integration

When the features are shared across multiple datasets while samples are collected from different cohorts or studies, the integration is typically referred to as horizontal integration. Despite the difference in scope, several general strategies for vertical integration may also be used for horizontal integration. For example, Bayesian multistudy factor analysis (De Vito et al. 2021) is an unsupervised dimension reduction approach analogous to those described in Section 2.1, but it identifies shared and unshared factors across studies rather than modalities. Multiple augmented RRR (Wang & Lock 2024) further decomposes covariate-driven and auxiliary structured variation that is shared or unshared across multiple sample cohorts. Joint and individual component regression (Wang et al. 2024) describe a supervised factor model to predict an outcome, with shared or unshared factors across multiple sample cohorts.

A few methods are designed for bidimensional integration across multiple modalities and sample cohorts or groups, combining vertical and horizontal integration as shown in Figure 1. The bidimensional factorization (BIDIFAC) method (Park & Lock 2020, Lock et al. 2022) considers linked data of the form $\left\{X_{kj}:p_{k}\times n_{j}\right\}$ for modalities k=1,…,K and sample groups j=1,…,J. The data are decomposed into lower-dimensional factors that are shared or unshared across both the modalities and the sample groups. Lock et al. (2022) applied BIDIFAC to “pan-omics pancancer” data, identifying factors that are shared or specific across four omics modalities (mRNA expression, miRNA expression, DNA methylation, and the proteome) and 29 cancer subtypes. Kallus et al. (2019) and Chen et al. (2022) describe alternative extensions of PCA that are applicable to bidimensionally linked (e.g., multiomic multicohort) data.

### 4.2. Longitudinal Analysis

An increasing number of studies are now collecting multiomics data longitudinally across multiple time points for the same individuals. This is essential for capturing dynamic biological processes such as disease progression and enables more nuanced investigations of causal molecular mechanisms. While most existing multiomics integration methods are designed for cross-sectional data, recent efforts have begun to extend these approaches to accommodate longitudinal settings. For instance, Wang & Wang (2024) proposed a multilayer exponential family factor model in which time-varying latent factors are used to capture disease trajectories from multimodal longitudinal data. They applied their model to Parkinson’s Progression Markers Initiative data to integrate heterogeneous clinical, cognitive, imaging, and molecular measures, uncovering lower-dimensional disease-related factors that capture the temporal neurodegeneration in Parkinson’s disease. Lu & Chandra (2024) introduced a nonparametric Bayesian method for clustering mixed-type, high-dimensional longitudinal data. Mallick et al. (2024) described a general framework for prediction from cross-sectional and longitudinal multiomics data, where a mixed-effects model is used to summarize temporal features, and individual-level random intercepts are extracted as predictors. Although these developments represent important progress, longitudinal multiomics integration remains a wide-open area with many unresolved methodological challenges and opportunities for future research.

### 4.3. Nonlinear Methods

Most of the methods reviewed thus far focus on linear combinations of omics features, thereby capturing only linear relationships. However, in practice, the underlying latent structures that carry important biological signals may reside in a much higher-dimensional space than that of the original data, and the relationships between omics predictors and outcomes may exhibit complex nonlinear patterns. In such cases, nonlinear methods can be particularly useful for uncovering more intricate structures and associations in multiomics data.

One of the most common strategies for extending linear methods to the nonlinear domain is through the use of kernels, often referred to as the kernel trick. The core idea is to replace the inner product in the original feature space with a kernel matrix, which implicitly computes the inner product in a higher-dimensional, often infinite-dimensional, feature space (Schölkopf & Smola 2002). This approach allows linear algorithms to be extended to nonlinear variants without explicitly computing the transformation.

For example, Bach & Jordan (2002) and Fukumizu et al. (2007) developed kernel CCA frameworks that identify the most correlated nonlinear transformations within a reproducing kernel Hilbert space. Tenenhaus et al. (2015) further extended this approach to accommodate more than two datasets. In the context of supervised learning, Zhao et al. (2018) introduced a kernel regression model that associates clinical outcomes with multiple omics modalities using a composite kernel. Little et al. (2025) later generalized this model to handle correlated samples. Additionally, multiple kernel learning frameworks have been developed for both supervised and unsupervised multiomics integration, where each omics modality is represented by one or more kernel matrices, and a weighted combination of these kernels is optimized for prediction or pattern discovery (Mariette & Villa-Vialaneix 2018, Yang et al. 2020, Briscik et al. 2024).

Despite their flexibility, nonlinear methods also present several limitations. Kernel-based approaches can be computationally expensive, particularly when applied to large-scale data with many samples, due to the need to compute and store large kernel matrices. Moreover, the mappings induced by kernel functions are implicit and often difficult to interpret, making it challenging to extract biologically meaningful insights from the learned features. Finally, selecting appropriate kernel functions for different omics modalities remains a nontrivial task and can significantly impact model performance.

### 4.4. Deep Learning Methods

Another burgeoning area in multiomics data integration is the application of DL algorithms. With their multilayered architectures, DL methods are well suited for modeling complex, nonlinear relationships and heterogeneous data structures. They are particularly popular in supervised learning tasks such as classification and prediction due to their strong empirical performance.

Autoencoders (AEs) are among the most widely used DL models for multiomics integration (Rumelhart et al. 1985). AEs consist of an encoder that maps input data to a latent space and a decoder that reconstructs the original input (Ma & Zhang 2018). They are often used for denoising, dimensionality reduction, and harmonizing modalities prior to downstream analysis. Numerous AE variants have been developed for multitask learning, feature representation, and survival prediction (Chaudhary et al. 2018, Zhang et al. 2021, Benkirane et al. 2023, Yao et al. 2024). Other DL models include generative approaches such as variational AEs, generative adversarial networks, and transformer-based models.

Despite their flexibility and predictive power, DL methods are often criticized for their lack of interpretability, high computational demands, and sensitivity to small sample sizes, which can lead to overfitting and poor generalizability. For comprehensive reviews, readers are directed to Kang et al. (2022) and Ballard et al. (2024).

## 5. REMAINING CHALLENGES AND FUTURE DIRECTIONS

### 5.1. Heterogeneity of Omics Data

Multiomics integration is complicated by the distinct data types and structures across different omics modalities. For example, sequencing-based data such as RNA-seq produce count data that, after normalization, can be approximately modeled by Gaussian distributions (Zhao et al. 2021). In contrast, microbiome data from 16S rRNA or metagenomic sequencing are compositional and reflect only relative abundances (Li 2015). Other technologies, such as mass spectrometry used in proteomics and metabolomics, yield continuous spectral data with varying scales (Chen et al. 2020).

A common strategy is to transform all data to a uniform type (e.g., continuous) and apply standard integration methods. However, this can lead to information loss and obscure biological interpretation. To address this, recent methods have been developed to directly handle mixed data types. Examples include penalized regression for mixed features (Yang et al. 2014, Zhang et al. 2017) and generalized factorization approaches for joint dimension reduction and association analysis (Li & Gaynanova 2018, Zhu et al. 2020).

In addition, many omics modalities offer auxiliary information that can enhance analysis, such as known pathways in gene expression or phylogenetic trees in microbiome data. Properly incorporating such structure can improve both accuracy and interpretability, though methods designed for specific modalities often lack generalizability.

Differences in data scale and dimension further complicate integration. For instance, RNA-seq may provide tens of thousands of features, while microbiome data typically include only hundreds. Measurements also differ in range: Methylation values are bounded between 0 and 1, while lipidomic data span much wider scales. Integrating data without adjusting for these differences may bias analyses toward high-dimensional or high-variance modalities, especially when regulatory relationships are not explicitly modeled. While preprocessing (e.g., filtering, normalization) helps mitigate imbalance, more principled solutions, such as latent variable models and multiple kernel methods, are increasingly used to align disparate modalities in a unified analytical framework.

### 5.2. Missing Data

Missing data are a common challenge in multiomics studies, as different modalities often vary in completeness across samples. A particularly prevalent issue is block-wise missingness, where entire omics layers are missing for subsets of individuals, often due to cost constraints or other sample-specific factors. For example, in the ongoing Genetic Epidemiology of Chronic Obstructive Pulmonary Disease (COPDGene) study (Regan et al. 2011), genomic, transcriptomic, proteomic, and metabolomic data were collected from over 10,000 participants. However, only 612 individuals have complete data across all four modalities.

Most existing integration methods assume complete data, limiting their applicability and statistical power in large-scale studies with extensive block-wise missingness. While a few approaches have been developed to impute missing omics blocks (Zhu et al. 2020, Xue & Qu 2021, Lock 2024), single-imputation methods may substantially underestimate uncertainty in downstream analyses (Samorodnitsky et al. 2024, Jiang et al. 2025). There is a pressing need for methods that both accommodate block-wise missingness across diverse integration tasks and adequately account for uncertainty through flexible multiple imputation frameworks that reflect the complex joint structure of multiomics data.

### 5.3. Incorporation of Prior Data

Most molecular features captured by omics technologies have been extensively studied across diverse biological contexts. As a result, there exists a rich body of prior data and domain knowledge relevant to most integrative analyses. For instance, numerous biological pathways and regulatory networks are known to persist across conditions. Traditionally, comparisons with prior knowledge are conducted post hoc through literature review or pathway enrichment analysis. Several tools have been developed for multiomics pathway enrichment (Kamburov et al. 2011, Hernández-de-Diego et al. 2018).

However, methods that directly incorporate prior knowledge into primary analytical tasks hold greater promise for improving both statistical power and biological interpretability. Recent advances include approaches that apply structured penalties to encourage similarity among features within predefined pathways or networks (Safo et al. 2022, Chekouo & Safo 2023). Bayesian frameworks also offer a natural way to encode prior information, for example, by specifying priors based on findings from past studies.

Despite their potential, integrating prior data presents challenges. These include the risk of incorporating bias, inconsistencies between studies, and the difficulty of selecting the most relevant and reliable sources of information. There remains a need for flexible, robust methods that can systematically leverage prior knowledge while accounting for its limitations.

### 5.4. Emerging Technologies

Ongoing advances in experimental technologies continue to drive new developments in multi-omics integration. In particular, single-cell and spatially resolved molecular profiling technologies are expanding the scope of integrative analyses by enabling data collection at unprecedented resolution. Reflecting their transformative potential, *Nature Methods* recognized single-cell multimodal omics as Method of the Year in 2019 (Teichmann & Efremova 2020), followed by spatially resolved transcriptomics in 2020 (Marx 2021).

These technologies introduce unique challenges for statistical integration. In single-cell experiments, molecular features (e.g., gene expression) are measured across thousands of cells per sample, resulting in data structures of the form $X_{ij}$: Features × Cells for sample i and modality j. Cells are typically not matched across samples or modalities, complicating cross-sample and cross-modality integration. Recent methods such as MoClust (Yuan et al. 2023) address this issue by clustering and annotating cell types across modalities, allowing cell-level data to be aggregated into comparable subtypes. This results in a multiway structure (Figure 1) where integration occurs across features, modalities, and cell subtypes. However, MoClust assumes all cells originate from a single sample, and in general, few methods exist for identifying shared cell types across both multimodal and multisample data.

Alternative approaches like MOFA+ (Argelaguet et al. 2020) perform unsupervised integration directly at the cell level. MOFA+ decomposes shared and modality-specific variation across multimodal single-cell data, similar in spirit to the methods discussed in Section 2.1 but with each cell considered a sample. While powerful, MOFA+ assumes that all modalities are measured on the same cells and does not scale easily to large cohorts where multimodal data may be partially observed across individuals.

The integrative analysis of spatially resolved multimodal data represents another emerging frontier. Many challenges parallel those of single-cell data, but with the added complexity that spatial relationships must be modeled explicitly. Incorporating spatial structure—e.g., through spatial smoothing or spatially aware clustering—is critical for accurate integration. Further challenges arise when modalities are measured at different spatial resolutions (e.g., single-cell resolution versus coarser aggregate regions), complicating direct alignment. More broadly, integrating data from diverse technologies, each with its own spatial and cellular granularity, presents substantial methodological opportunities. This area is rapidly evolving and is likely to become increasingly central to multiomics research.
